# Supplementary material for: MetaRibo-Seq measures translation in microbiomes
Source: Nat Commun. 2020 Jun 29;11:3268. doi: 10.1038/s41467-020-17081-z (PMC7324362; doi:10.1038/s41467-020-17081-z)
Supplement: Supplementary file 10 — Supplementary Data 7 [file 41467_2020_17081_MOESM10_ESM.zip › File2/Confidence_High_Taxonomy/368046_out.krona.html]

Javascript must be enabled to view this page.

members
magnitude
magnitudeUnassigned
count
unassigned
taxon
rank

368046\_out

5

2
superkingdom
5

5
phylum
976

5
200643
class

5
171549
order

171552
family
5

5
genus
838

1

SRS012849\_contig\_number\_11930
2293125
species

species
2292365

SRS012969\_contig\_number\_3619SRS023526\_contig\_number\_32768SRS077849\_contig\_number\_20327
3

species

SRS015794\_contig\_number\_4121
2292054
1
